# The first case of bipolar transurethral resection of the prostate for giant prostatic hyperplasia: A case report

**DOI:** 10.1097/MD.0000000000032455

**Published:** 2022-12-23

**Authors:** Deheng Cui, Guoqiang Chen, Jianbin Luo, Minjie Zhang

**Affiliations:** a Department of Urology, The Second Hospital of Longyan, Fujian, China.

**Keywords:** bipolar transurethral resection of the prostate, giant prostatic hyperplasia

## Abstract

Rationale: We admitted an 89-year-old male patient diagnosed with benign prostatic hyperplasia and a prostate volume of approximately 522 ml measured by magnetic resonance imaging.

Patient concerns: He had previous hypertension, diabetes, and coronary artery disease.

Diagnoses: Giant prostatic hyperplasia.

Interventions: We opted for bipolar transurethral resection of the prostate (BTURP).

Outcomes: The urinary flow rate was detected 1 week after surgery, with a peak flow rate of 17 mL/s and a voided volume of 156 mL. The follow-up was 11 months, with an international prostate symptom score of 7 and a quality of life (QOL) of 1.

Conclusion: Complete removal of the prostate is not our goal; reducing the risk of surgery and improving the quality of life of the patient is the theme.

## 1. Introduction

**Figure 1.**
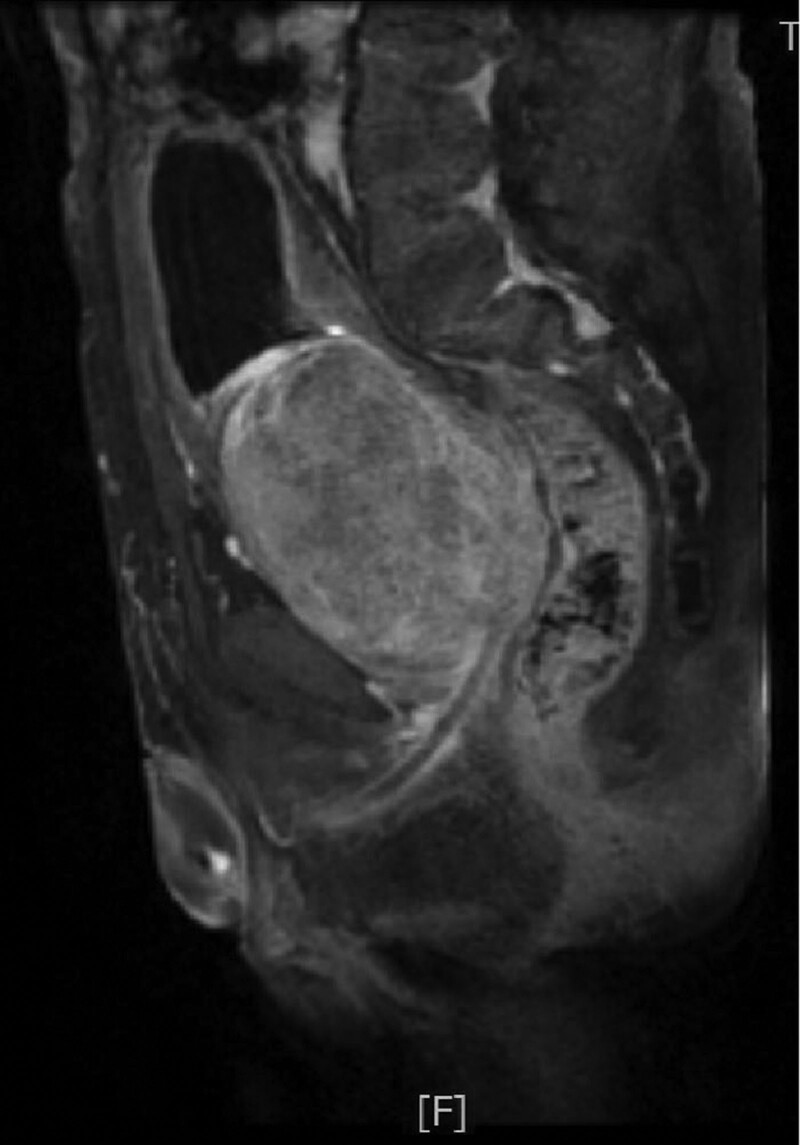
Sagittal plane of magnetic resonance image, huge prostate pressing on the rectum and bladder shifting upward.

**Figure 2.**
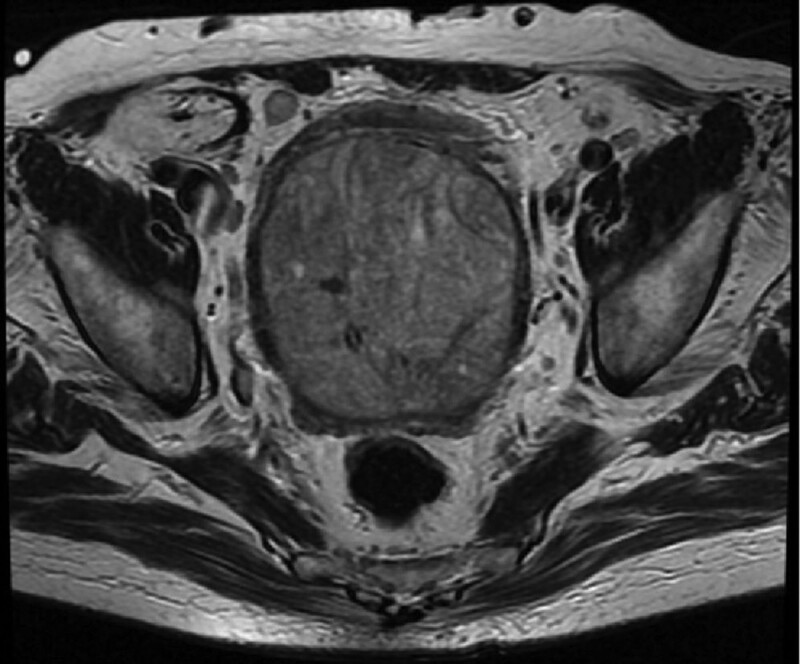
Cross-section of magnetic resonance image, huge prostate filling the pelvic cavity.

We admitted an 89-year-old male with complaints of progressive dyspareunia for ten years, long-term oral finasteride and tamsulosin hydrochloride extended-release capsules, international prostate symptom score of 25 and quality of life (QOL) of 5; acute urinary retention developed 1 month ago, and a catheter was left in place to drain the urine until now. He had a total serum prostate specific antigen of 28.01 ng/mL, a prostate size of 9.50 cm × 9.60 cm × 11.00 cm measured by magnetic resonance imaging, and an estimated volume of 522 mL (Figs. 1 and 2). He had previous hypertension, diabetes, and coronary artery disease, which were well controlled by long-term outpatient follow-up treatment.

The patient refused to undergo prostate puncture biopsy and requested surgery to relieve lower urinary tract symptoms and get rid of the catheter. Usually, open prostatectomy (OP) is chosen, but open surgery has high bleeding and mortality^[1]^; the patient had many underlying diseases and was close to 90 years old. To decrease the risk of surgery and to meet the patient’s expectations, we decided to perform bipolar transurethral resection of the prostate (BTURP). The patient was operated on with intralesional anesthesia, and the operation time was 130 minutes; 110 g of tissue was removed, and the postoperative hemoglobin decreased by 18 g/L. The catheter was left in place for 3 days and then removed. Pathological results suggested benign prostatic hyperplasia. The urinary flow rate was detected 1 week after surgery, with a peak flow rate of 17 ml/s and a voided volume of 156 mL. The follow-up was 11 months, with an international prostate symptom score of 7 and a QOL of 1.

## 2. Discussion

Benign prostatic hyperplasia is very common in older men, and its incidence increases with age. 1993 Fishman and Merrill first defined a prostate mass greater than 500 g as “giant prostatic hyperplasia.”^[2]^ To date, only 31 cases could be retrieved from the literature, including 1 case of observation and follow-up,^[3]^ 1 case of a long-term indwelling catheter, 1 case of prostatic artery embolization, 1 case of laparoscopic prostatectomy, and 27 cases of open prostatectomy (3 deaths, 11.11%).^[4]^ To the best of our knowledge, this is the first case reporting BTURP for giant prostatic hyperplasia with satisfactory results. Advanced age is no longer a contraindication to BTURP,^[5]^ and a long-term follow-up study found a 3% versus 3.4% reoperation rate ten years after BTURP versus OP, which is not a significant difference^[6]^; and BTURP is gradually replacing OP for the treatment of large volume prostatic hyperplasia. Preoperatively, the patient’s physical health was fully assessed and operated on by an experienced urologist, which needs to take into account the insufficient length of the operating instruments. In this patient, the electrode was extended to reach the innermost edge of the prostate at the longest point.

## 3. Conclusions

Complete removal of the prostate is not our goal; reducing the risk of surgery and improving the QOL of the patient is the theme.

## Author contributions

All authors edited, reviewed and approved the manuscript.

**Conceptualization:** Deheng Cui.

**Investigation:** Guoqiang Chen.

**Methodology:** Guoqiang Chen, Jianbin Luo, Minjie Zhang.

**Resources:** Jianbin Luo.

**Writing – review & editing:** Deheng Cui.
